# Facile Construction of a Solely-DNA-Based System for Targeted Delivery of Nucleic Acids

**DOI:** 10.3390/nano11081967

**Published:** 2021-07-30

**Authors:** Ziwen Dai, Juan Li, Yongfang Lin, Zhigang Wang, Yang Huang

**Affiliations:** 1 College of Materials Science and Engineering, Shenzhen University, Shenzhen 518055, China; 2 Key Laboratory of Optoelectronic Devices and Systems of Ministry of Education and Guangdong Province, College of Optoelectronic Engineering, Shenzhen University, Shenzhen 518060, China; 3 School of Pharmaceutical Sciences, Health Science Center, Shenzhen University, Shenzhen 518055, China; lijuan150515@163.com (J.L.); 2018224041@mail.szu.edu.cn (Y.L.)

**Keywords:** DNA nanostructure, DNA aptamer, targeted drug delivery

## Abstract

We designed a functional drug delivery system based solely on DNA. The whole system was built with only four DNA strands. Cyclization of DNA strands excluded the formation of byproducts. DNA aptamers were equipped to endow triangular DNA nanostructures with targeting ability. The homogeneity of materials enabled not only facile construction but also convenient loading of nucleic acid-based drugs with much ease.

## 1. Introduction

Macromolecule-based drugs represent a class of important and efficacious therapeutics which are used to treat a variety of diseases, such as diabetes, immunological disorders and cancers. Many of those drugs have been developed, and some have been approved for clinical applications, such as monoclonal antibodies [1], inactivated viruses-based vaccines [2] and nucleic acids [3]. However, two obstacles still restrict their universal clinical applications. The first one originates from their biological nature which includes intrinsic instability [4] and immunogenicity, and the second one is related to the administration process: biological membranes create solid barriers for drug penetration [5]. Thus, macromolecular drugs usually depended on drug delivery systems to provide protection and to cross biological membranes. Recently, among various drug delivery systems [6,7,8], DNA nanostructures have emerged as powerful and ideal drug delivery carriers due to their predictability and programmability in fabrication [9], high stability in biological environments [10] and low cytotoxicity and immunogenicity [11]. In fact, DNA nanotechnology has developed into a prosperous field, and a variety of DNA nanostructures have been built for diverse applications, such as tracking [12], imaging [13] and delivery [14]. On the other hand, targeted delivery could increase the availability of drugs and decrease the adverse effects; however, the acquisition of targeting ability for drug carriers usually involves conjugation with targeting groups [15]. This is especially difficult for macromolecules because of the low coupling efficiency and discouraging purification process [16]. Thus, a targeting group that can be integrated into drug carriers without much effort would be an attractive choice. Interestingly, one class of DNA has been found to possess targeting abilities; they are called DNA aptamers. DNA aptamers are short synthetic oligonucleotides which can fold into tertiary structures and bind to their targets with high affinity and high specificity. Their targets include small molecules, proteins and cells [17]. Many DNA aptamers have been screened for targeting cell membranes, and they have similar performances in targeting compared with protein-based antibodies while bypassing their drawbacks. Thus, it is quite attractive and also facile to construct a targeted drug delivery system with building blocks entirely made of DNA, as DNA aptamers can be integrated seamlessly into DNA nanostructures.

In this work, we report the design and fabrication of a solely-DNA-based drug delivery system which was built upon a DNA nanostructure (the platform) and had a DNA aptamer integrated for targeting purposes. Specifically, a triangular DNA nanostructure was synthesized and integrated with the DNA aptamer AS1411 that targeted nucleolin, which is over-expressed on the membranes of many cancer cells [18]. The system demonstrated significantly enhanced stability in biological environments and could penetrate into nucleolin-abundant cancer cells without the assistance of transfection reagents. Furthermore, as a proof-of-concept study, an antisense oligonucleotide (ASO) that can silence *c-myc* was loaded onto the system, and it was found that the system was able to downregulate gene expression at both mRNA and protein levels with satisfactory efficacy. Considering its simple design and facile fabrication process, we believe our system can be further improved to expand its scope of applications, and that it represents an economic and effective approach for delivery of nucleic acid-based drugs.

## 2. Materials and Methods

### 2.1. Materials and Instrumentation

Boric acid, glacial acetic acid, tris(hydroxymethyl)aminomethane (Tris), ethylenediaminetetraacetic acid disodium salt (EDTA), magnesium chloride hexahydrate, acrylamide, *N*,*N*′-methylenebisacrylamide, urea, ammonium persulfate, *N*,*N*,*N*′*N*′-tetramethylethylenediamine, lithium perchlorate, cyanogen bromide, formamide, acetone, acetonitrile, 2-(*N*-mophorlino)ethanesulfonic acid monohydrate (MES) and glycerin were purchased from J&K Chemicals Ltd. (Beijing, China) and used as received. Glass wool (silanized) and Stains-All were purchased from Sigma-Aldrich (Shanghai, China). Sephadex G-25 medium was purchased from Beijing Solarbio Science & Technology Co., Ltd. (Beijing, China). All oligonucleotides were purchased from Sangon Biotech (Shanghai, China) Co., Limited. TransZol Up Plus RNA Kit, TransScript All-in-One First-Strand cDNA Synthesis SuperMix for qPCR kit and PerfectStart Green qPCR SuperMix kit were purchased from TransGen Biotech Co., Limited (Beijing, China). 6X DNA loading dye, Lysotracker Green (DND-26) and Hoechst 33,342 were purchased from Thermo Fisher Scientific Inc. (Shanghai, China). Cell Counting Kit-8 was purchased from Dojindo Laboratories. Anti-*c-Myc* antibody [Y69] and anti-GAPDH antibody were purchased from Abcam PLC (Shanghai, China). Anti-rabbit IgG HRP-linked antibody #7074 was purchased from Cell signaling Technologies (Shanghai, China). Fetal bovine serum (FBS), phosphate buffered saline (PBS), Dulbecco’s modified eagle’s medium (DMEM), penicillin–streptomycin solution and trypsin were purchased from Invitrogen (Shanghai, China). 1X TAMg buffer was composed of 45 mM Tris and 7.6 mM MgCl_2_ with pH adjusted to 7.8 using glacial acid. 1X TBE buffer was composed of 90 mM Tris, 90 mM boric acid and 1.1 mM EDTA with pH adjusted to 8.0 using hydrochloric acid. All aqueous solutions were prepared in ultrapure water supplied by Millipore purification system (Shanghai, China).

In vitro fluorescence measurements were conducted on Edinburgh Instruments Fluorescence Spectrometer FS5 (Edinburgh, England).

Gel electrophoresis experiments were carried out on a 20 × 20 cm vertical electrophoresis apparatus (JY-SCZ6+, Beijing Junyi, Beijing, China).

The sequences of all DNA strands used in the present research are summarized in Table 1.

### 2.2. Synthesis of Cyclic Single-Stranded DNA T

The procedure for the synthesis of cyclic single-stranded T was adapted from the protocols reported in our previous research. Generally, 24 nmoles of CTS and 24 nmoles of Tt were lyophilized and re-dissolved in 300 μL of MES buffered solutions (2-(*N*-mophorlino)ethanesulfonic acid, 250 mM, pH = 7.6), respectively, and cooled to 0 °C on ice. Then the solutions were mixed and incubated on ice for another 30 min. After that, 600 μL of cyanogen bromide solution (5M in dry acetonitrile) was added to the reaction mixture and vortexed for 30 min on ice, which was followed by adding 12 mL of LiClO_4_ solution (2%w/vin acetone). The mixture was incubated on dry ice for 1 h and then centrifuged at 14,000 rpm and 4 °C for 10 min. After disposing of the supernatant, the pellet was dried and re-dissolved in 120 μL of autoclaved H_2_O and separated by 12% denaturing PAGE gels. The desired band was sliced and collected, and the target product DNA was extracted and desalted by Sephadex G25 (Solarbio Science, Beijing, China). The quantification was conducted on Implen Nanophotometer N60 (Germany).

### 2.3. Preparation of Triangular-Shaped DNA Nanostructures (TDNs)

For different purposes, different TNDs were assembled with different DNA strand combinations. Generally, DNA strands of equal molar quantities were lyophilized together and re-dissolved in 1X PBS buffer. The mixture was then heated to 60 °C and kept for 5 min, followed by a slow annealing to 4 °C over1 h. The component strands for each assay are summarized in Table 2.

### 2.4. Fetal Bovine Serum Digestion Assay

The self-assembled DNA nanostructures (TDNs) were mixed with stock FBS solutions to make final 10% FBS solutions containing 1.915 μM of the DNA nanostructure. These samples were incubated at 37 °C for 0, 1, 2, 4, 8, 12 or 24 h. For denaturing gel analysis, the samples were denatured by being heated at 60 °C for 20 min with 95% formamide. Denatured samples were then characterized on 12% denaturing PAGE gel and stained with Stains-All, and the images were analyzed with ImageJ.

### 2.5. Confocal Fluorescence Imaging and Flow Cytometry Analysis

A549 lung cancer cells were seeded in confocal dishes (2 × 10^5^ cells) and incubated for 48 h. For localization experiment, Cy3-labeled DNA nanostructures (AS1411-TDN) were added to the dishes and incubated for another 24 h. For in vivo FRET studies, Cy3/C5-dually labeled TNDs were used. The cells were treated with Lysotracker Green DND-26 (2 h prior to imaging) and Hoechst 33342 (30 min prior to imaging), respectively and then washed with PBS buffer. The imaging was performed on a Zeiss LSM 880 confocal fluorescence microscope. The Cy3 fluorophore was excited with 543 nm laser lines and the emission spectrum was collected from 550 to 600 nm for Cy3.

For flow cytometry analysis, A549 lung cancer cells were seeded in 35 mm cell culture dishes, and when they reached a confluence of 90%, Cy5-labeled DNA nanostructures (AS1411-TDN) were added to the dishes. The cells were left grown for 2, 4, 6or 8 h, and then collected and analyzed on an Attune N×T acoustic focusing cytometer.

### 2.6. Western Blot and Quantitative Polymerase Chain Reaction (qPCR) Assay

A549 cells were seeded in 35 mm cell culture dishes (2 × 10^5^ cells) and incubated for 48 h. Then the cells were incubated with 30 μL cell culture medium containing 9.58 mM AS1411/c-myc-TDNs for 48 h. Cells treated with PBS buffer were used as control. Then the cells were washed with PBS and protein lysates were extracted using RIPA buffer. Protein quantification was performed on an Epoch Microplate Spectrophotometer. Then, 50 μg of proteins were separated using SDS-PAGE with Bio-Rad Mini-Protean Tetra System and then electroblotted to nitrocellulose membranes in transfer buffer. After that, the nitrocellulose membranes were blocked with 10% (*w*/*v*) non-fat milk power in TBS-T buffer and incubated with anti-c-myc antibodies overnight at 4 °C, rinsed and incubated in horseradish peroxidase-conjugated secondary antibody for 1 h. The signal was detected using an enhanced chemiluminescence kit and imaged by a chemiluminescence system (ChemiScope series 5300 (Shanghai, China)).

For the qPCR assay, A549 cells were seeded in 35 mm cell culture dishes (2 × 10^5^ cells) and incubated for 48 h. Then the cells were incubated with 30 μL cell culture medium containing 9.58 mM AS1411/c-myc-TDNs for 48 h. Cells treated with PBS buffer were used as controls. After 48 h of incubation, the cells were washed with PBS and total mRNA was isolated using the Trizol reagent. The cDNA was obtained from samples with thecDNA Reverse Transcription Kit. Then the cDNA was used as a template for qPCR amplification according to the protocol. The expression of the target gene was normalized to β-actin. The result was processed into relative quantification with a delta-delta Ct method using the cells treated with PBS buffer as the control (see Supplementary Materials for the primer sequences).

## 3. Results

**Figure 1:**
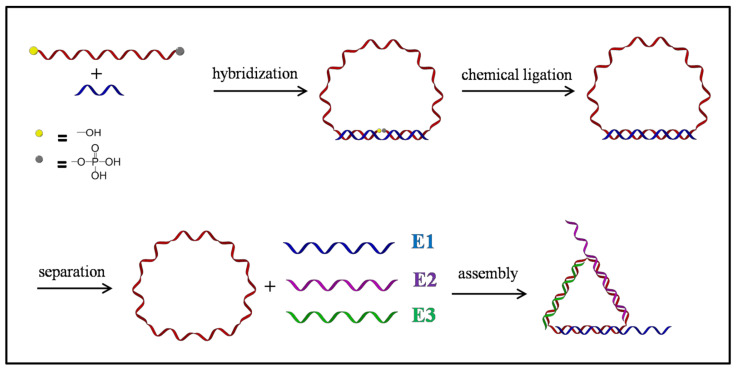
Schematic demonstration of the synthesis of the cyclic DNA strand and self-assembly of the all-DNA system.

As shown in Figure 1, the system was built upon a cyclic single-stranded DNA and prepared by stepwise self-assembly.

As was reported, capping the open ends of DNA strands could effectively increase their resistance to enzymatic degradation and enhance their biological stability [19,20]. In our design, the two open ends were capped simultaneously by cyclization. The cyclic DNA strand T was synthesized through DNA-templated chemical ligation, purified by polyacrylamide gel electrophoresis (PAGE) and confirmed by both denaturing PAGE and high-resolution matrix-assisted laser desorption-ionization time of flight (MALDI-TOF) mass spectrometry [21].

**Figure 2:**
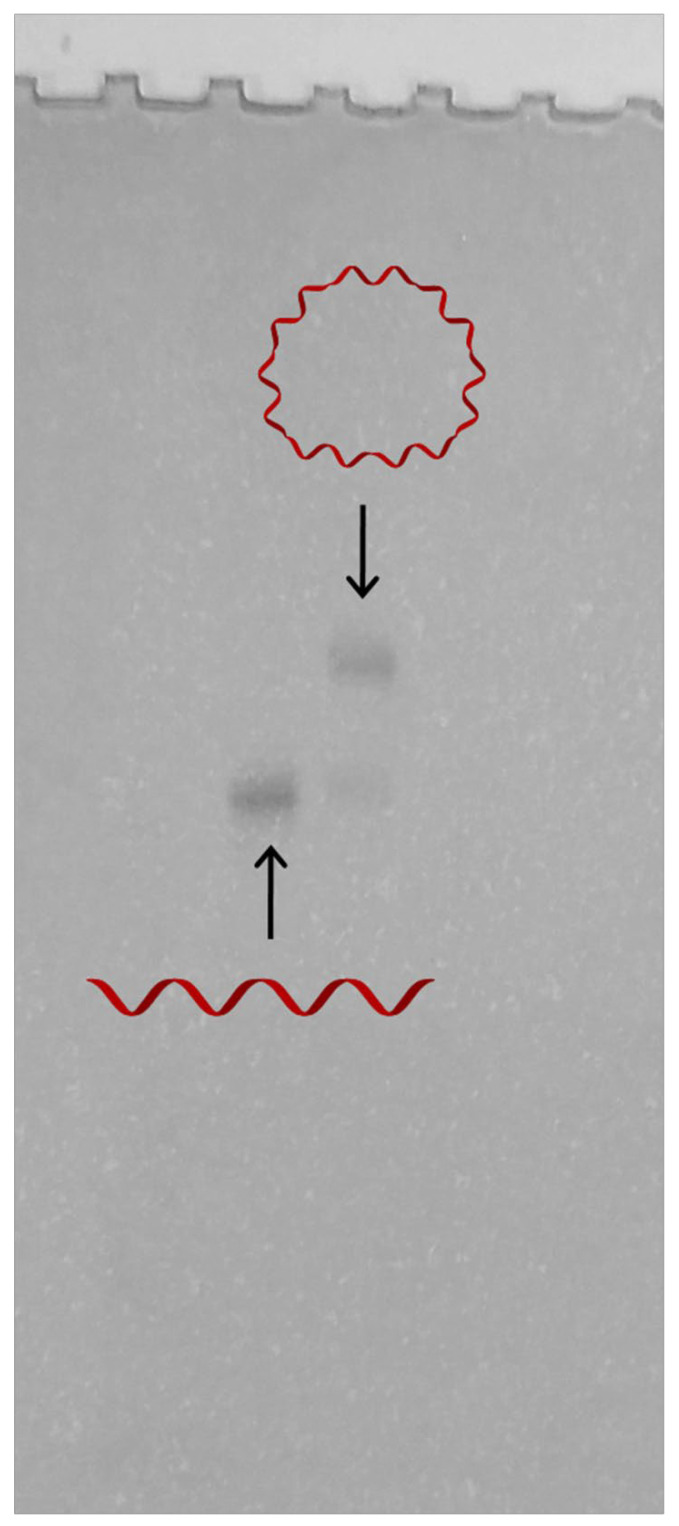
Denaturing PAGE analysis of the synthesized cyclic DNA strand T.

Cyclic DNA strand T showed retarded mobility in electrophoresis compared with its linear counterparts, and this was demonstrated by denaturing PAGE (Figure 2).

**Figure 3:**
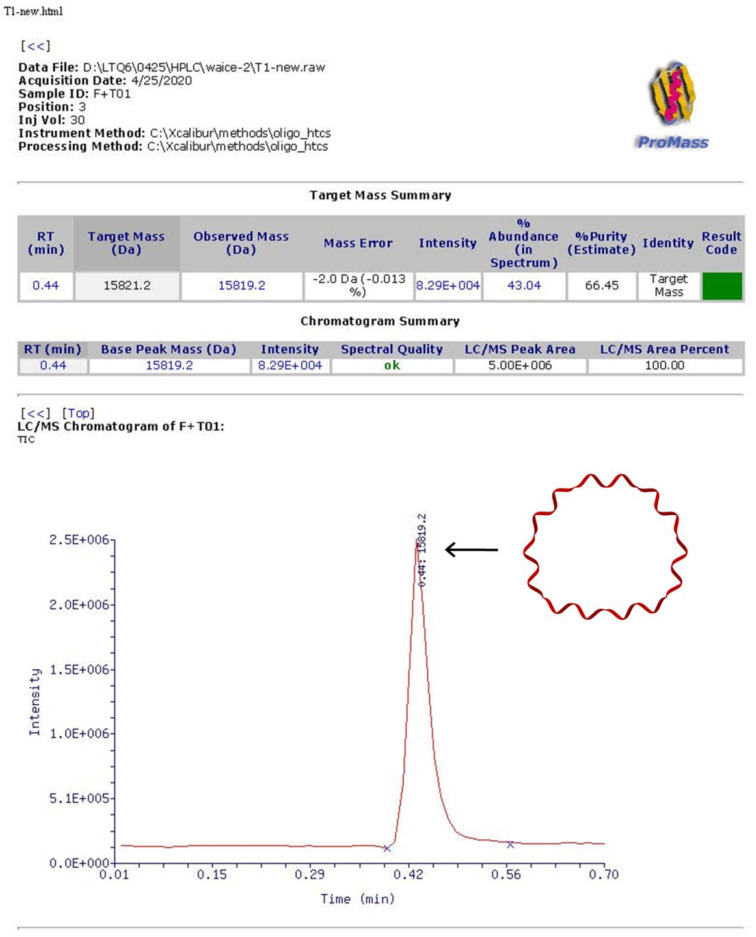
MALDI-TOF mass spectrometry analysis of the synthesized cyclic DNA strand T. Calculated: 15,821.15 g/mol; found: 15,819.2 g/mol.

Strong evidence came from the high-resolution mass spectrometry, which undoubtedly confirmed that the desired products were obtained (Figure 3).

**Figure 4:**
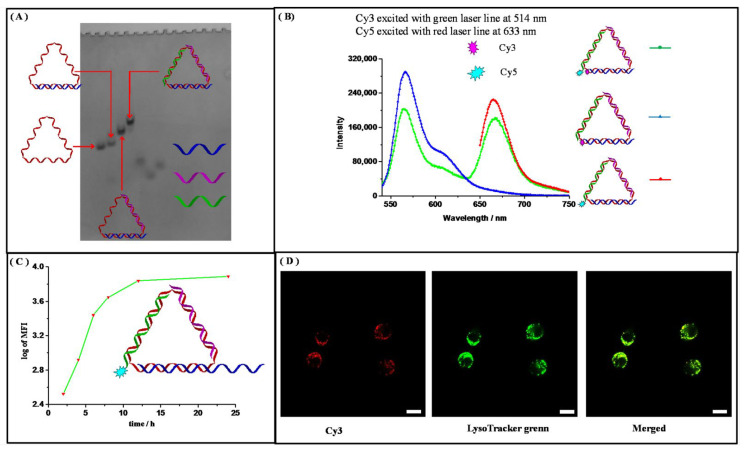
The self-assembly of the solely DNA-based drug delivery system and its cellular uptake byA549 cells. (**A**) Non-denaturing PAGE analysis of the stepwise assembly of the triangular DNA nanostructure. (**B**) In vitro FRET confirmation of the formation of desired DNA nanostructure. Excited at 514 nm for Cy3 and 633 nm for Cy5. (**C**) Flow cytometry analysis of the cellular uptake of the AS1411-integrated DNA nanostructure in A549 cells. (**D**) Confocal fluorescence images of AS1411-integrated DNA nanostructures incubated with A549 cells. The scale bar is 25 μm.

**Figure 5:**
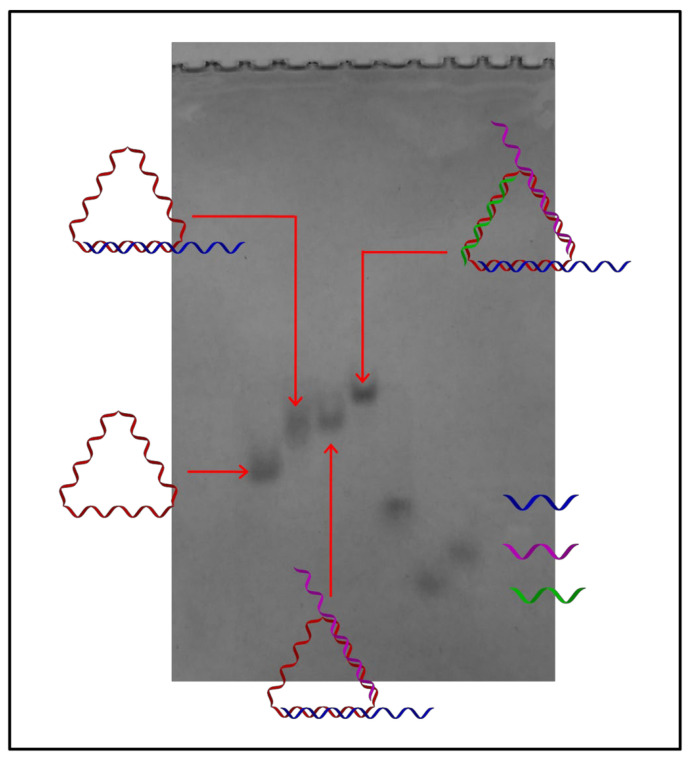
Non-denaturing PAGE analysis of the stepwise assembly of AS1411/c-myc-TDN.

After that, the construction of the triangular DNA nanostructures (TDN) was realized by stepwise self-assembly, which was demonstrated by non-denaturing PAGE (Figure 4A and Figure 5).

**Figure 6:**
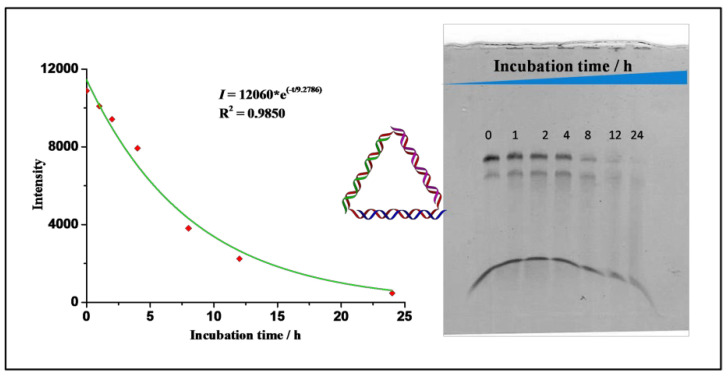
Denaturing PAGE analysis of the bare TDNs in FBS digestion test at different time points.

Cyclization of template DNA excluded the formation of undesired byproducts, which could not have been avoided if the strand were open-ended. This was especially advantageous compared with those three-way-junction-based (3WJ) DNA nanostructures, as our TDNs could be prepared by one-pot assembly without generating undesired byproducts [22]. The successful formation of the desired structure was also evidenced by an in vitro fluorescence resonance energy transfer (FRET) experiment in an aqueous solution (Figure 4B). Cy3 and Cy5 fluorophores were anchored in close proximity to each other on the DNA nanostructure, and when Cy3 was excited, the fluorescence from both Cy3 and Cy5 could be detected only when the designed structure was formed. Together, they confirmed that the designed DNA nanostructure TDN was successfully formed with high efficiency. Next, we tested the stability of the TDNs. It was reported that DNA nanostructures could enhance their overall stability compared to the single-stranded components [23]. We tested their stability in simulated biological environments through a fetal bovine serum (FBS) digestion assay. Each TDN was incubated with 10% FBS solutions for different time intervals and then analyzed via denaturing PAGE. From the results, we calculated that the half-life for our primary TDN was 6.43 h (Figure 6), and this is in accordance with the results reported previously by Lo and coworkers [24].

**Figure 7:**
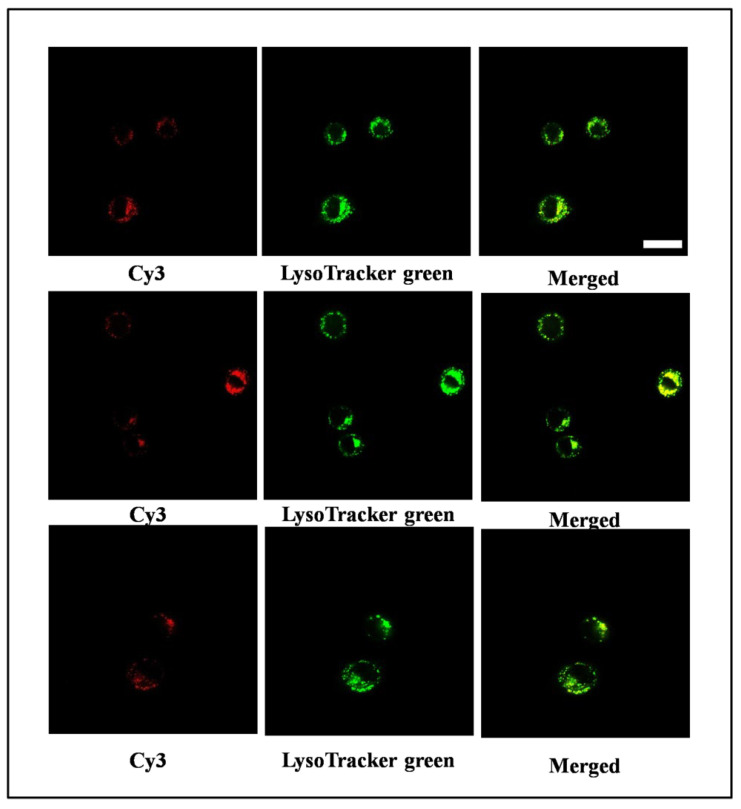
More confocal fluorescence images for intracellular localization of a Cy3-labeled DNA nanostructure in A549 cells. The scale bar is 25 μm.

As these DNA nanostructures would be used for delivery purposes, the above results encouraged us to explore their cell-penetrating abilities. Here, the DNA aptamer AS1411 was extended from a one-component DNA strand and loaded onto the nanostructure by one-pot self-assembly to obtain the AS1411-integrated TDNs (AS1411-TDN). We first tested its binding to the target cell A549, which over-expressed nucleolin, by flow cytometry. It was found that, after about 8 h, the binding of the AS1411-TDNs to A549 cells almost reached saturation (Figure 4C). To monitor the behavior of our system after penetrating into cells, AS1411-TDN was modified with Cy3 fluorophore and imaged with a confocal laser microscope. Co-localization experiments showed that the DNA nanostructures mainly accumulated in lysosomes (Figure 4D and Figure 7), which indicates that they probably entered cells through an endocytosis pathway [25].

**Figure 8:**
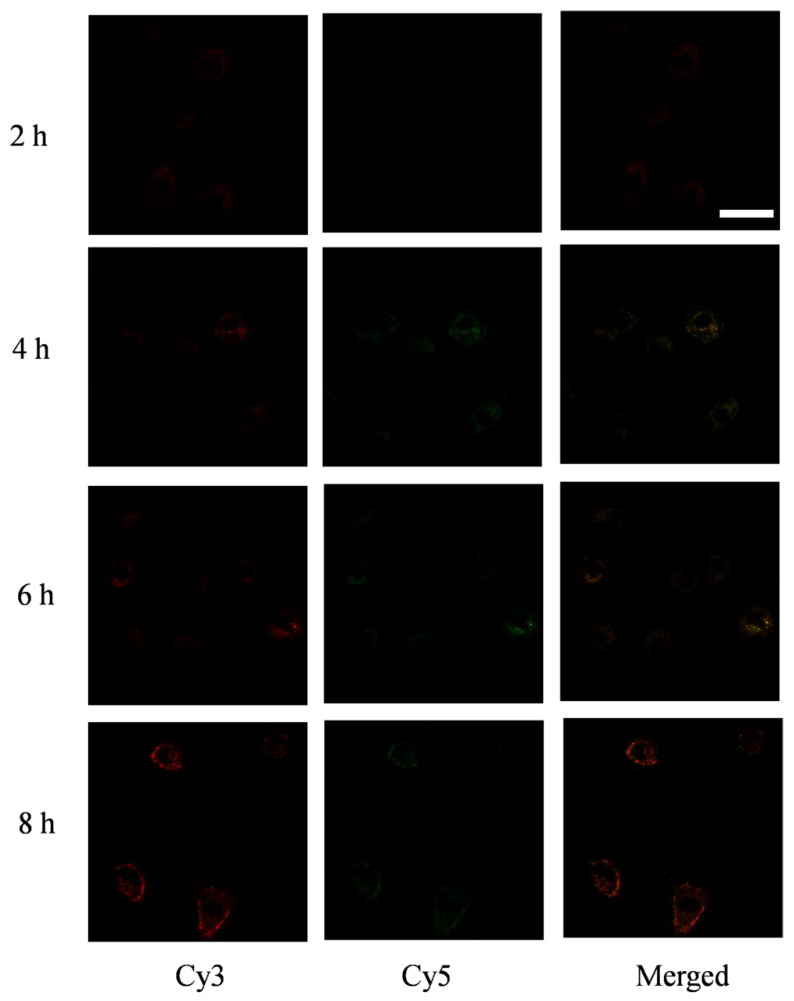
FRET studies of doubly-labeled DNA nanostructures in A549 cell lines. Samples were excited with a 514 nm laser and emissions were collected from 550 to 600 nm (green channel for Cy3) and from 650 to 700 nm (red channel for Cy5). The scale bar is 25 μm.

Notably, AS1411-TDN crossed cell membranes without the assistance of transfecting reagents. We attributed this to the installation of DNA aptamers. The binding of DNA aptamers to their targets on cell membranes facilitated the endocytosis of the DNA nanostructures, which is especially attractive for drug delivery. Meanwhile, *in-cell* FRET was also performed. Interestingly, the *in-cell* FRET experiment demonstrated that the fluorescent signal from Cy5 experienced a rise-then-fall process, which indicated that our DNA system began to collapse due to the highly acidic environment inside endosomes (Figure 8) [26].

The signal reached its peak at about 6 h, which is in good agreement with the result from the FBS assay. Together, the above results demonstrate that our DNA-based drug delivery system possesses the potential to take nucleic acid-based cargoes through cell membranes.

**Figure 9:**
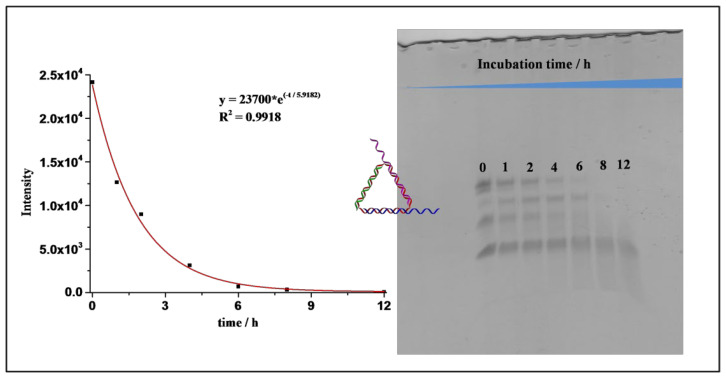
Denaturing PAGE analysis of the aptamer and ASO-containing TDNs in the FBS digestion test at different time points.

As the system was built solely with DNA strands, it would be especially facile to use it to deliver nucleic acid-based drugs, such as antisense oligonucleotides (ASOs), siRNAs, mRNAs and PNAs. They could be loaded onto the system either by direct integration as component strands (DNAs) or by complementary hybridization (RNAs and PNAs). As a test, we examined the ability of the DNA system to deliver a piece of ASO that silenced the *c-myc* gene to manipulate gene expression. ASOs can bind to mRNAs and induce their degradation by cellular machinery, thereby suppressing the expression of specific genes [27]. The DNA nanostructure was loaded with the AS1411 DNA aptamer and the *c-myc* ASO to make a DNA warship (AS1411/c-myc-TDN). The DNA warship was subjected to the FBS digestion test first, and it was found that the half-life of the DNA warship was slightly lower, at 4.1 h (Figure 9).

This shortened half-life was attributed to the extended DNA overhangs being more susceptible to enzymatic degradation, causing the whole system to have a shortened half-life in the FBS test.

**Figure 10:**
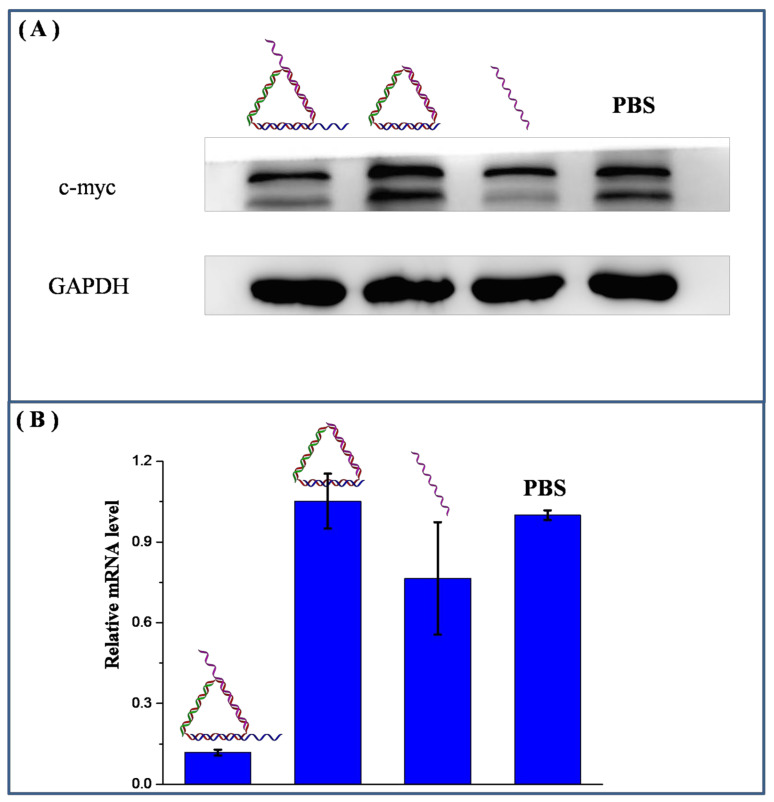
Suppression of c-myc gene expression induced by DNA warship. (**A**) Western blot analysis of c-myc protein expression levels in A549 cells after treatment with DNA warship, bare DNA nanostructure, free ASO or PBS. (**B**) Relative mRNA levels in A549 cells after treatment with DNA warship, bare DNA nanostructure, free ASO or PBS.

Next, the DNA warship was incubated with A549 lung cancer cells for evaluation of gene silencing efficiency. A Western blot assay [28] indicated that the DNA warship effectively inhibited the expression of *c-myc* at the protein level, even at very low concentrations (Figure 10A). From the result we can see that the intensity of lower bands of the c-myc bands decreased in intensity as the DNA warship and single-stranded ASO groups did the opposite. This probably indicates that the ASO sequence we used targeted a specific site of the target gene. The concentration of the *c-myc* sequence in the DNA warship was only 1/20 of that of the single strands. However, the DNA warship demonstrated satisfactory efficacy comparable to the single-stranded DNA control group, even if the latter was administered in large quantities. This demonstrated the advantages of our DNA warship-based system—that is, it can greatly decrease the concentrations of ASO drugs that need to be used, even though the system is quite simple. Meanwhile, a quantitative reverse-transcriptase polymerase chain reaction (qRT-PCR) was also carried out and proved that the DNA warship more effectively suppressed the expression of *c-myc* genes at the mRNA level compared with all other control groups (Figure 10B). This again demonstrated the advantages of our DNA warship-based system. It should be noted that the bare DNA nanostructures without an antisense oligonucleotide sequence showed negligible effects in both experiments, which indicates that our DNA nanostructures were bio-orthogonal and safe for biomedical applications. Based on the above results, we believe that our DNA-based drug delivery system could be also used for the delivery of other types of nucleic acid-based drugs, such as siRNAs.

## 4. Discussion

Various DNA nanostructures have been applied in drug delivery, such as DNA origami-based nanostructures [28]. Compared with our system, DNA origami suffered from some disadvantages, such as the use of whole genome of bacteriophage M13mp18 and hundreds of DNA strands, which was quite costly. Meanwhile, assembly of a DNA origami was accompanied by the generation of fragmented products which usually posed challenges for separation and purification. Thus, our system outperformed the DNA origami by its low cost and high yield. On the other hand, tetrahedral DNA nanostructures were once the most frequently used drug delivery carriers [29]. However, in addition to the byproduct problem, they needed a fast heating-cooling cycle which might be adverse for loading of temperature-sensitive cargoes, whereas our system can be prepared at room temperature. Thus, we believe our system has unique advantages compared with other DNA nanostructures. Of course, there is still room for improvement in the properties of our system, which will be priorities of ongoing work.

## 5. Conclusions

In summary, we have designed and prepared a simple DNA nanostructure-baseddrug delivery system which is equipped with a DNA aptamer for targeting and is loaded with an antisense oligonucleotide sequence for gene expression regulation in cancer cells. The system showed good stability and high biocompatibility, and significantly downregulated the expression of the target gene at both mRNA and protein levels. Most importantly, our system was built with only four DNA strands, which were also cheap. Considering the facile complexation of drugs with carriers, our system was quite labor-saving compared with other drug delivery systems, such as polymers and gold nanoparticles. We believe our DNA warship system can be further improved and expanded for other applications.

## Figures and Tables

**Table 1:** Oligonucleotide sequences used in this project. Cy3 represents cyanine 3 fluorophore, Cy5 represents cyanine 5 fluorophore.

Primer	Sequence (5′–3′)
CTS	TCGGTAGATTGATAGCCCAGATCGGTTAGAGTAATCTCTTGATTTGAGCAC/3′ Phosphate/
Tt	TCTACCGAGTGCTCA
E1	TCTACCGAGTGCTCA
E2	TCAAGAGATTACTCT
E3	CCGATCTGGGCTATC
E1-5′ Cy3	/Cy3/TTCTACCGAGTGCTCA
E2-5′ Cy5	/Cy5/TTCAAGAGATTACTCT
E3-3′ Cy5	CCGATCTGGGCTATCT/Cy5/
E2-c-myc	AACGTTGAGGGGCATTTTCAAGAGAGGACTCT
E3-5′ Cy3	/Cy3/TCCGATCTGGGCTATC
E2-3′ Cy5	TCAAGAGATTACTCTT/Cy5/
E1-AS1411	GGTGGTGGTGGTTGGGTGGTGGTGGTTCTACCGAGTGCTCA

**Table 2:** The DNA strand combinations for assembling different TDNs used in different experiments.

Experiment	Strand Combinations
FBS assay	T, E1, E2, E3
Flow cytometry assay	T, E1-AS1411, E2, E3-Cy5
FRET assay	T, E1-Cy3, E2, E3-Cy5
Confocal imaging assay	T, E1-AS1411, E2, E3-Cy3
Western blot assay	T, E1-AS1411, E2-c-myc, E3
RT-qPCR assay	T, E1-AS1411, E2-c-myc, E3

## Data Availability

Data available in a publicly accessible repository.

## Supplementary Materials

The following are available online at https://www.mdpi.com/article/10.3390/nano11081967/s1, Figure S1: The schematic demonstration of DNA nanostructures with different strand combinations.
