# Supplementary material for: Facile Construction of a Solely-DNA-Based System for Targeted Delivery of Nucleic Acids
Source: Nanomaterials (Basel). 2021 Jul 30;11(8):1967. doi: 10.3390/nano11081967 (PMC8398070; doi:10.3390/nano11081967)
Supplement: Supplementary file 1 [file nanomaterials-11-01967-s001.zip › nanomaterials-1270717-supplementary.pdf]

nanomaterials

Supporting information

Facile Construction of a Solely-DNA-Based System for Targeted Delivery of Nucleic Acids

Ziwen Dai [1,2,*], Juan Li [3], Yongfang Lin [3], Zhigang Wang [3,*] and Yang Huang [1,*]

[1] College of Materials Science and Engineering, Shenzhen University, Shenzhen 518055, China
[2] Key Laboratory of Optoelectronic Devices and Systems of Ministry of Education and Guangdong Province, College of Optoelectronic Engineering, Shenzhen University, Shenzhen 518060, China
[3] School of Pharmaceutical Sciences, Health Science Center, Shenzhen University, Shenzhen 518055, China; lijuan150515@163.com (J.L.); 2018224041@mail.szu.edu.cn (Y.L.)
* Correspondence: dai-ziwen@foxmail.com (Z.D.); wangzg@szu.edu.cn (Z.W.); y.huang@outlook.com (Y.H.)

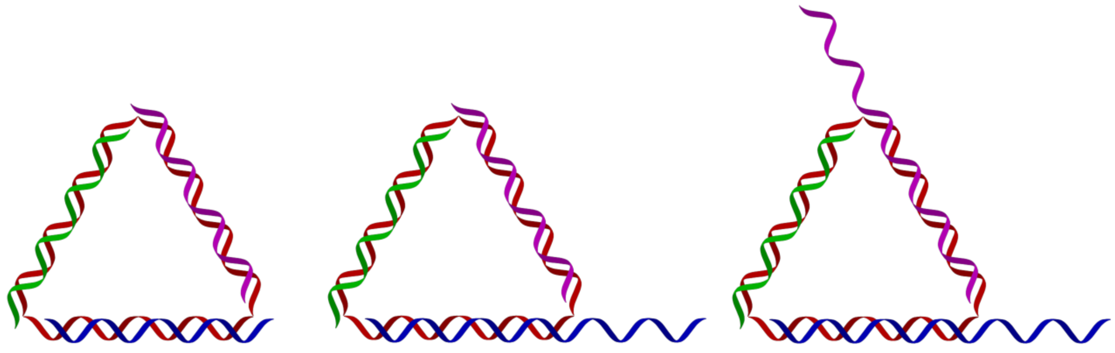

Figure S1. The schematic demonstration of DNA nanostructures with different strand combinations.

1. Fetal Bovine Serum Digestion Assay

To determine the half-life of the DNA nanostructure, the band at time point 0 h was identified and used as the baseline to remove the background intensity. The decay rate λ and half-life $t_{1/2}$ was derived as follows:

$$I = I_0 e^{-\lambda t}$$

$$\tau = 1/\lambda$$

$$t_{1/2} = \tau \ln 2$$

where τ is time constant, I_0 is initial band intensity at time point 0 h and I is the band intensity at time t.

Thus, the half-life of our DNA nanostructure was:

$$t_{1/2} = 9.2786 * \ln 2 = 6.43 \text{ h}$$

Following the same protocol, we conducted the same experiment for the aptamer- and ASO-containing DNA warship, and calculated that the half-life $t_{1/2}$for the DNA warship was 4.10 h.

Compared with the bare TDNs, the aptamer- and ASO-integrated DNA nanostructures showed a slightly reduced half-life in the FBS digestion test. This was attributed to the fact that the extended DNA overhangs were more susceptible to enzymatic degradation and thus the whole system had a shorter half-life.

2. Western Blot and Quantitative Polymerase Chain Reaction (qPCR) Assay

For qPCR assay, A549 cells were seeded in 35 mm cell culture dishes (2×10^5 cells) and incubated for 48 h. Then, the cells were incubated with 30 μL cell culture medium containing 9.58 mM AS1411/c-myc-TDNs for 48 h. Cells treated with PBS buffer were used as control. After 48 h incubation, the cells were washed with PBS and total mRNA

was isolated using the Trizol reagent. The cDNA was obtained from samples through cDNA Reverse Transcription Kit. Then, the cDNA was used as template for qPCR amplification according to the protocol. The expression of the target gene was normalized to β-actin. The result was processed into relative quantification with a delta–delta Ct method using the cells treated with PBS buffer as the control. The primer sequences used in the analysis were listed as follows:

c-myc forward: 5′- GTGGCACCTCTTGAGGACCT-3′

c-myc reverse: 5′-TGGTGCTCCATGAGGAGACA-3′

β-actin forward: 5′-GCATCCTGTCGGCAATGC-3′

β-actin reverse: 5′-GTTGCTATCCAGGCTGTGC-3′
